# Using the Candidacy Framework to understand individual, interpersonal, and system level factors driving inequities in women with breast cancer: a cross-sectional study

**DOI:** 10.1038/s44276-024-00103-4

**Published:** 2024-10-23

**Authors:** Mar Estupiñán Fdez. de Mesa, Afrodita Marcu, Emma Ream, Katriina L. Whitaker

**Affiliations:** School of Health Sciences, University of Surrey, Research Park, Guildford, Surrey GU2 7YH UK

## Abstract

**Background:**

Persistent inequities in breast cancer outcomes exist. Understanding women’s experiences along the care pathway is the first step to finding solutions to tackle these inequities.

**Methods:**

Secondary data analysis of the 2017/2018 English National Cancer Patient Experience Survey (*n* = 25,408) using logistic regression to explore inequities in care experience by sociodemographic factors (age, ethnicity, socioeconomic position, sexual orientation) across 59 survey questions. We used the Candidacy Framework to interpret and organise our findings.

**Results:**

Compared to older (65–74) and White British women, young (35-44, OR = 0.55 [0.44, 0.69]), Asian (OR = 0.52 [0.41, 0.67]), Black (OR = 0.67 [0.46, 0.97]) and White Other (OR = 0.63 [0.49, 0.81]) women were more likely to rate their overall care experience less positively, respectively. Similar findings were observed along all domains of the cancer pathway. Through a candidacy lens, we identified multilevel factors related to this variation including prolonged help-seeking behaviours (individual), poor patient-provider communication (interpersonal), and variation in access to healthcare professionals and resources (system level).

**Conclusion:**

Multilevel factors influence inequities in the experience of care along the breast cancer pathway for young women and women from minoritised groups. Interventions are necessary to ensure cancer care systems are responsive to women’s health needs and provide equity of care to all patients.

## Introduction

Despite innovations and advancements in cancer care, minoritised women (individuals that have experienced marginalisation due to their gender, race/ethnicity, or social background [1]) continue to experience inequities in breast cancer care and outcomes [2]. Evidence suggests that their unequal location in society – linked to the intersection of multifaceted identities with the social determinants of health and power structures, influences women’s opportunities to seek and receive prompt breast cancer care [3, 4]. Moreover, discriminative policies and practices have been associated with inadequate provision of cancer services and diminished quality of care [5]. Racism and other forms of discrimination shape how patients interact with breast cancer services, access treatment and resources, and influence care experience and outcomes for women from minoritised populations [4, 6].

To address these challenges and close the equity gap in breast cancer care and outcomes, a multisectoral approach is necessary [4]. Researchers can contribute by generating evidence from different geopolitical contexts to enhance our collective understanding of factors driving inequities in breast cancer [7]. This evidence is essential to inform policies and services and to provide optimal and sustainable breast cancer care to all women [8]. A first step to achieving this goal is to understand the multilevel factors that operate when women navigate care services and to map pathways to breast cancer outcomes through examining cancer patient care experience [9].

Patient care experience is a process indicator that captures the quality of care received [10] and this has been positively associated with clinical effectiveness and patient safety [11]. Patient care experience has become an indicator of quality in healthcare in many countries and it is often used to inform cancer quality improvement plans [12–14]. In the UK, cancer patient experience is collected annually through the National Cancer Patient Experience Survey (NCPES) [12] and their outcomes analysed – for instance, by comparing breast cancer to all cancers, and comparing women to men. Results from earlier NCPES studies suggest that patients with breast cancer are more likely to rate their care more positively compared to all other patients with cancer [15]. Moreover, when compared to men, women with cancer (all tumours) are more likely to rate their care less positively [16].

What is less clear is how care experience may differ between and within women with breast cancer. This gap in the literature needs addressing to ensure services respect personal needs and preferences and to facilitate equitable and prompt access to care for all women [4]. We sought to address this gap by specifically focusing on women with breast cancer accessing a publicly funded national health service in England – that is, a healthcare system based on equity, universal, and free at the point of care [17]. Using self-reported feedback collected through the national survey our aim was three-fold: (i) to describe and summarise patterns of inequities in care experience by sociodemographic factors (age, ethnicity, socioeconomic position, sexual orientation) across all survey questions; (b) to identify and interpret multilevel factors driving differences in breast cancer care experience; and (c) to map findings across the breast cancer care pathway.

## Material and methods

### Data source

Secondary data analysis was performed on anonymous national survey data obtained for non-for-profit research from the UK Data Service [18]. The survey data was collected by Quality Health (UK) on behalf of the National Health Service (NHS). Patients (aged 16 and over) were included in the survey sample if they had a primary diagnosis of cancer from any hospital in England [12]. Non-respondents were followed up with two reminders.

The NCPES survey [19] is a nationally validated tool that contains 59 multiple-choice and yes/no questions covering cancer patients’ experience across the care pathway including presentation to services, diagnostic tests, treatment decisions, communication with doctors and nurses, involvement of Clinical Nurse Specialists (CNSs), inpatient and outpatient care, home care, support, and follow up after discharge (Table S1). The survey outcomes are summarised using binary responses (categorised as positive/negative) to inform quality improvement plans. Uninformative responses (e.g., ‘Prefer not to say’/’I don’t know) are excluded. We used these binary outcomes to examine the variation in care experience between women with breast cancer. Evidence suggests that minority ethnic groups are under-represented in national surveys [20]. To address the challenge this poses for this research, we used two consecutive, identical annual surveys (2017, 2018). The survey data is collected annually over 3 months, and the risk of duplications is low. The datasets were anonymised at source, therefore ethical approval was not required to conduct a secondary analysis of these national data.

**Fig. 1:**
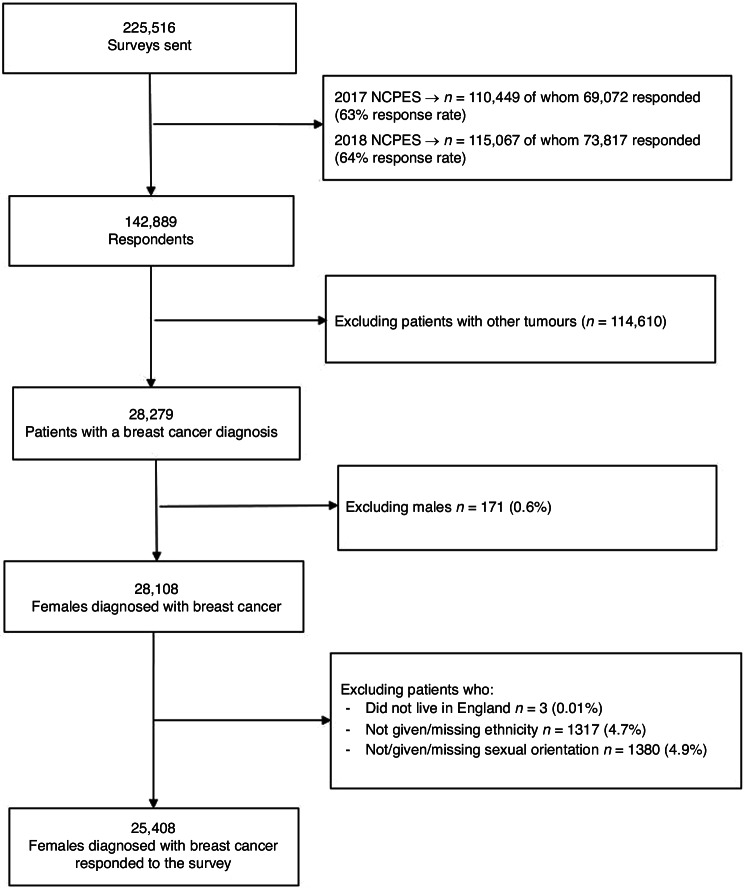
Flow diagram of the number of respondents included in the analysis. Number of 2017/2018 NCPES respondents included at each stage of the analysis.

A summary of sociodemographic variables is included in Supplementary Table S2. The eligible population was women (female hospital record defined as sex assigned at birth) with a primary diagnosis of breast cancer (*n* = 25,408) (Fig. 1). In line with other studies using the same datasets and to enable comparison, age was categorised into six groups (16–34, 35–44; 45–54; 55–64; 65–74; ≥75). Self-reported ethnicity was the chosen measure for ethnicity because it is considered to be the ‘gold standard’ [21]. A UK six-group ethnic classification (White British, Other White, Asian, Black, Mixed, and Other ethnicities) [22] was used in the analysis. Socioeconomic data was not included in the 2017/2018 datasets; however, respondents’ deprivation score (Index of Multiple Deprivation (IMD) quintiles) was available. In the absence of individual socioeconomic data, we used the IMD as a proxy measure. The IMD quintiles (values 1 to 5, 1= least affluent) is a validated measure of socioeconomic position and widely used in healthcare research [23, 24], and therefore it was deemed appropriate for this study. Due to the small sample size, we recoded self-reported sexual orientation into a binary variable (i.e., heterosexual and sexual minority groups). Comorbidities were excluded from the analysis due to the high proportion of missing data ( > 5%). Analyses were restricted to respondents from whom full sociodemographic data were available (i.e., age, ethnicity, deprivation, sexual orientation). Non-resident patients who were referred to English NHS Trusts for treatment were excluded (*n* = 3).

### Statistical analysis

First, we performed descriptive analyses to summarise respondents’ characteristics (frequency/percentage) and the unadjusted percentage distribution of overall positive care experience responses for each survey question. Subsequently, for each question, we fitted a mixed-effect logistic regression model with positive/negative experience as the dependent variable. We controlled for age group, ethnicity, deprivation, and sexual orientation because these independent variables have been previously associated with patient care experience [16, 25, 26]. A random effect for the hospital patients attended was also included in the model to account for the cluster effect of concentration of patients with different characteristics (e.g., ethnicity, deprivation) across different hospitals. For six questions (q.1, q.3, q.5, q.39, q.52, q.59) the model suggested there was no variation for the random effect. For these questions, fixed-effect logistic regression models were used. No control for multiple comparisons was made. Findings from logistic regression analyses were reported using Odds Ratios (ORs) and 95% Confidence Intervals (CIs); *p*-values < 0.05 were deemed significant. All analyses were performed using SPSS Statistics 28.0.1 software.

Based on a similar approach [15], patterns of inequity across the breast cancer pathway were visually summarised for each question. For each one, we used a red-amber-green colour coding convention (red=most negative experience; green=most positive experience) and compared each subcategory to the reference group, reporting ORs and 95% CI. Colour coding was applied using MS Excel Office 16 software. Subsequently, we used the Candidacy Framework [27, 28] to interpret and organise our findings as explained in the next section.

### Candidacy Framework

The Candidacy Framework describes the process by which patients define and understand what interventions are appropriate for them, and how their perceived care experience influences their engagement with services [27]. The Candidacy Framework can be used to understand the equity of care and to interpret the multiple factors (individual, social contexts, and organisational factors) influencing the receipt of healthcare [27].

The framework has seven overlapping stages: identification of candidacy, navigation, the permeability of services, appearances at health services, adjudications, offers and resistance, and operating conditions (Table 1). These stages are related to how patients may perceive themselves to be entitled to receive services (*identification of candidacy*), the ease by which participants can navigate services (*permeability of the healthcare system*), how professionals’ judgement may influence their decisions to offer services (*adjudication of services*), and logistical challenges in the system (*operation and local production*). The framework has been used in healthcare research [28–31]; however, it has been under-utilised in the study of breast cancer inequities.

**Table 1:** Adapted description of the stages of the Candidacy Framework to the breast cancer pathway [27, 29].

Stages	Definition	Adapted description stages
1. Identification of candidacy	“Process by which individuals come to view themselves as legitimate candidates for particular services”	The process by which breast cancer patients come to view themselves as legitimate candidates for particular services.
2. Navigation of services	“Knowing how to make contact with appropriate services in relation to candidacy”	Knowledge of services available at different stages of the breast cancer journey and ability to access services. Some determinants that may hinder or prevent access are transport costs, time costs, convenient appointment times, and availability of language translation services.
3. Permeability of services	“Includes the level of explicit and implicit gate-keeping within a service and the complexity of its referral systems; in addition it refers to the ’cultural alignment’ between users and services”	Ease with which breast cancer patients can access services. Includes levels of gatekeeping within service, the complexity of the referral system, and the ‘cultural alignment’ of services with women’s needs and values.
4. Appearance at services	“The work that individuals must do to assert their candidacy in an interaction with a health care professional”	Breast cancer patients’ ability to assert their candidacy by presenting at breast cancer services, articulating their issues, and articulating their ‘need’ for care.
5. Adjudication by healthcare professionals	“Candidacy as expressed by service-users is validated or otherwise by healthcare professionals and this influences subsequent offers of services”	A person’s candidacy is judged by healthcare professionals, subsequently influencing the person’s progression through services and access to care. Adjudication may disadvantage certain breast cancer patients by perceiving them as either ‘deserving’ or ‘undeserving’.
6. Offers of/resistance to services	“Emphasises that follow-up services may be appropriately or inappropriately offered and that these may or may not be acted upon by service-users”	Breast cancer patients may refuse offers at multiple stages in their journey to treatment including resisting offers for treatment (e.g., referral, treatment, reconstruction).
7. Operating and local production	“This incorporates factors that influence decisions about subsequent service provision (e.g., the resources available for addressing candidacy) and the kinds of contingent relationships that develop between professionals and service-users over a number of encounters”	Incorporates factors at societal and macro levels that influence candidacy, such as the availability of local resources for addressing candidacy, and the relational aspects that develop between the healthcare provider and patient over multiple visits.

In this study, we used the Candidacy Framework as a critical theoretical tool to guide a more sophisticated and structured interpretation of the survey findings. Through a candidacy lens, we moved beyond the compartmentalisation of the cancer pathway in clinical terms (e.g., prevention, diagnosis, treatment, survival) [32] to focus on the multilevel and intertwined factors influencing experience along the continuum of care. The advantage of using this approach is that we could focus on the patient journey as a means of elucidating their perspectives and how these may relate to the wider system to influence breast cancer outcomes [33].

**Fig. 2:**
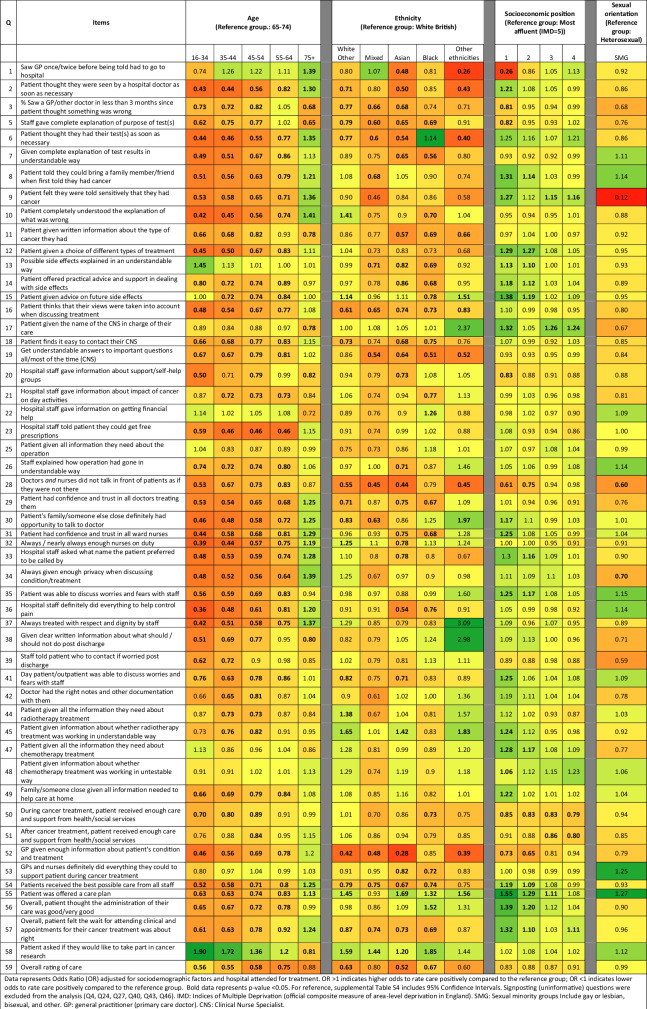
Association between respondents’ socio-demographic characteristics and cancer patient experience.

**Fig. 3:**
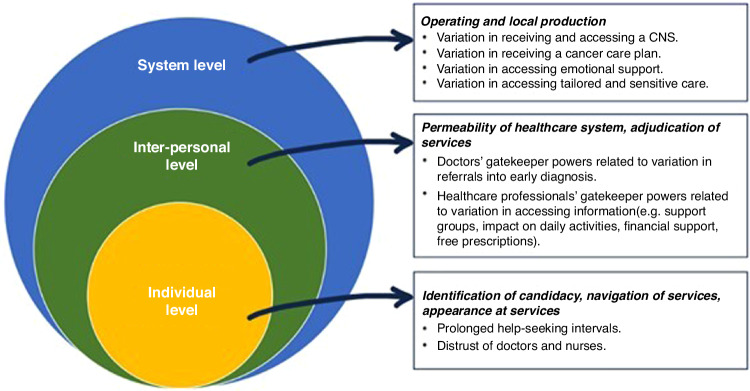
Multilevel summary of breast cancer inequities according to the Candidacy Framework.

**Fig. 4:**
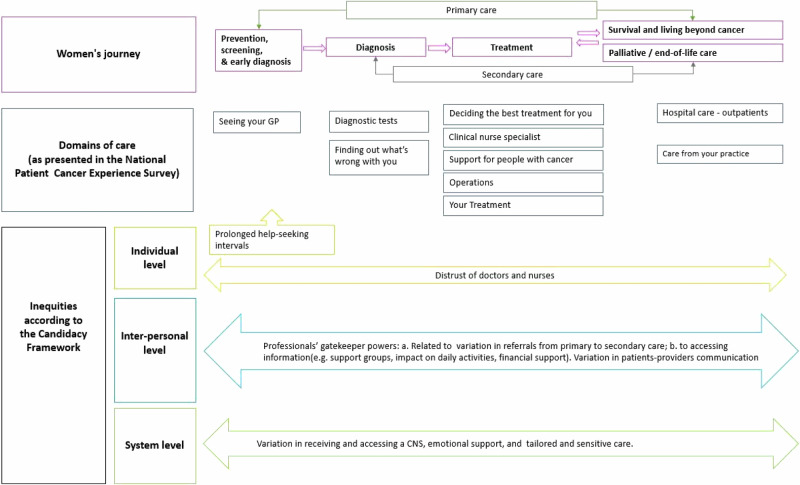
Illustration of the breast cancer pathway and main points of contact with primary and secondary care, the national survey categories by domain of care, and inequities according to the Candidacy Framework.

In practice, we first adapted the seven stages of the framework to the breast cancer pathway (Table 1). Then, the first author mapped each survey question according to the stages of the Candidacy Framework (as defined in Table 1) and discussed and resolved discrepancies with the research team (Supplementary Table S3). Guided by Table S3, we assessed our findings by domain of care and question (Fig. 2) and identified patterns of inequity and common themes emerging across the survey. Next, we elucidated how these findings related to individual, interpersonal, and system-level factors (Fig. 3) and how these could explain the observed differences in experience among women with breast cancer. Finally, we mapped findings along the cancer pathway (Fig. 4).

## Results

In total, 142,889 patients completed the 2017/2018 NCPES surveys. Of these, 25,408 female respondents had a primary breast cancer diagnosis and full valid sociodemographic data (age, ethnicity, deprivation, and sexual orientation) (Fig. 1).

### Descriptive statistics

Table 2 and Supplementary Table S1 show the totals and percentage distribution of the sample characteristics and number of positive responses by question. Most participants were older (aged 65–74 years, 28.6%), White British (89.5%), from most affluent backgrounds (least deprived, 25.3%), and heterosexual (98.7%). The percentage distribution of positive responses ranged from 32.1% (q58 - patient asked if they would like to take part in cancer research) and 37.9% (q55 – was the patient offered a written plan) to 96.9% (q25 – patient given all information they need about their operation).

**Table 2:** Socio-demographic characteristics of survey respondents.

Characteristics	All	%
Age		
16–34	400	1.6
35-44	1758	6.9
45–54	5621	22.1
55–64	6775	26.7
65–74	7259	28.6
75+	3595	14.1
Ethnicity		
White British	22,729	89.5
Other White	1082	4.3
Mixed ethnicity	222	0.9
Asian	806	3.2
Black	437	1.7
Other ethnicity	132	0.5
Socioeconomic position		
5 (Most affluent)	6439	25.3
4	6043	23.8
3	5554	21.9
2	4362	17.2
1 (Least affluent)	3010	11.8
Sexual orientation		
Heterosexual	25,065	98.7
Sexual minority groups	343	1.3

Ethnic group based on UK ONS categories [69]. Socioeconomic position: Official composite measure of area-level deprivation (Indices of Multiple Deprivation (IMD)). Sexual minority groups include gay or lesbian, bisexual, and others.

### Adjusted analysis

Table 3 shows the overall rating of care experience by sociodemographic factors. The full model containing all predictors was statistically significant, X2(138, *N* = 24,742) = 299.193, *p* < .001, suggesting that the model was able to distinguish between respondents who rated their care experience positively versus those who did not. Compared to the older reference group (65–74 years), findings showed that younger respondents were more likely to rate their overall care experience less positively (35–44 years, OR_adj,_ = 0.55 [0.44, 0.69]). Among minority ethnic groups, respondents from Asian (OR_adj,_ = 0.52 [0.40, 0.67]), Black (OR_adj,_ = 0.67 [0.46, 0.97]), and White Other (OR_adj,_ = 0.63 [0.49, 0.81]) descendent were more likely to rate their overall care less positive than White British. No statistically significant differences were identified among respondents from differing social backgrounds and sexual orientations.

**Table 3:** Survey question 59: Overall rating of care experience. Socio-demographic variation.

Characteristic	Unadjusted odds of reporting a positive overall care experience			Odds adjusted for patient factors and hospital of treatment		
	OR	95% CI	*p-value*	OR	95% CI	*p-value*
**Age**						
16–34	0.50	(0.33, 0.76)	**<0.001**	0.56	(0.37, 0.86)	**0.01**
35–44	0.49	(0.36, 0.61)	**0.00**	0.55	(0.44, 0.69)	**<0.001**
45–54	0.54	(0.46, 0.64)	**<0.001**	0.58	(0.49, 0.69)	**<0.001**
55–64	0.73	(0.62, 0.87)	**<0.001**	0.75	(0.63, 0.89)	**<0.001**
65–74	Reference					
75+	0.89	(0.72, 1.10)	0.27	0.88	(0.71, 1.09)	0.23
**Ethnicity**						
White British	Reference					
White Other	0.58	(0.46, 0.73)	**<0.001**	0.63	(0.49, 0.81)	**<0.001**
Mixed ethnicity	0.66	(0.38, 1.15)	0.14	0.80	(0.46, 1.40)	0.44
Asian	0.43	(0.34, 0.55)	**<0.0001**	0.52	(0.40, 0.67)	**<0.001**
Black	0.54	(0.38, 0.78)	**<0.0001**	0.67	(0.46, 0.97)	**0.04**
Other ethnicity	0.50	(0.27, 0.92)	**0.03**	0.60	(0.32, 1.13)	0.12
**Socioeconomic position**						
5 (Most affluent)	Reference					
4	0.88	(0.74, 1.05)	0.16	0.91	(0.76, 1.08)	0.27
3	0.84	(0.70, 1.00)	**0.04**	0.87	(0.72, 1.04)	0.11
2	0.79	(0.66, 0.95)	**0.01**	0.88	(0.72, 1.06)	0.18
1 (Least affluent)	0.71	(0.58, 0.87)	**<0.001**	0.83	(0.67, 1.03)	0.09
**Sexual orientation**						
Heterosexual	Reference					
Sexual minority groups	0.79	(0.50, 1.24)	0.31	0.99	(0.62, 1.57)	0.96

*OR* Odds Ratio. OR > 1 indicates higher odds of rating care experience positively compared to the reference group, OR < 1 indicates lower odds of rating care experience positively compared to the reference group. *CI* Confidence Interval. Bold data represents *p*-value < 0.05. Socioeconomic position: Correspond to Indices of Multiple Deprivation (an official composite measure of area-level deprivation in England). *SMG* Sexual minority groups Include gay or lesbian, bisexual, and others.

Figure 2 and supplemental Table S4 show the association between patient individual sociodemographic characteristics and cancer patient experience by survey question. In summary, younger respondents (aged ≤65) and respondents from Asian, Black, and Mixed ethnicities reported less favourable care experience across all domains of care more often than any other age and ethnic groups, while older patients (aged 75 + ) reported comparatively more positive care experiences. Similarly, sexual minority groups reported less favourable care experience across most domains compared to heterosexual respondents. However, this was not always statistically significant. Respondents from the least affluent backgrounds reported more positive care experiences than any other group in this category. However, there were specific questions where the least affluent respondents reported less favourable care experience than any other group (e.g., q.1 – saw GP once/twice before being told to go to the hospital; q.20 – hospital staff gave information about support/self-help groups; q.28 – doctors and nurses did not talk in front of patients as if they were there, and q.52 – GP [primary care doctor] given enough information about patient’s condition and treatment). Below, we summarised by theme the most salient findings observed across all survey questions that are amenable to intervene to improve outcomes for women with breast cancer.

#### Help-seeking behaviours

Help-seeking behaviours varied across groups. Data suggest younger females (particularly aged 35–44 (OR_adj,_ = 0.71 [0.60, 0.86])), Asian (OR_adj,_ =  0.68 [(0.54, 0.86]), and the least affluent (OR = 0.81 [0.68, 0.96])) more often sought help more than 3 months after they thought something was wrong with them (prolonged help-seeking interval).

#### Access to services and healthcare professionals

Doctors were identified as gatekeepers for referrals to secondary care. For instance, Asian (OR_adj,_ = 0.48 [0.30, 0.65]), other ethnicities (OR_adj,_ = 0.26 [0.15, 0.46]), and the least affluent females (OR_adj,_ = 0.26 [0.15, 0.46]) more often reported they had to see their doctor three or more times before being referred to secondary care. Similarly, healthcare professionals were identified as gatekeepers to accessing a variety of support resources that are relevant to patients’ journeys. For instance, there was variation in receiving information about support groups (young women (16–34) OR_adj,_ = 0.50 [0.37, 0.68], Asian (OR_adj,_ = 0.73 [0.58, 0.91]), and least affluent (OR_adj,_ = 0.83 [OR_adj,_ = 0.70, 0.98] were less likely to receive this information) and about the impact of cancer on day activities (young women (35–44) (OR_adj,_ = 0.72 [0.65, 0.82]) and Black women (OR_adj,_ = 0.77 [0.58, 0.91]) less likely to receive this information). Clinical Nurse Specialists (CNSs) are patients’ first point of contact and therefore they play a fundamental role in cancer care [34]. However, in this study we found that patients encountered difficulties to access their CNSs, particularly young (young women (16–34) OR_adj,_ = 0.66 [0.50, 0.88]), Asian (OR_adj,_ =  0.68 [0.56, 0.82]), Black (OR_adj,_ =  0.75 [0.57, 0.97]), and White other respondents (OR_adj,_ = 0.73 [0.62, 0.87]).

#### Patients-healthcare providers communication

Our findings showed patterns of inequities related to patient-provider communication across all domains of care. This ranges from variation in receiving information about treatment (e.g., younger respondents were less likely to receive the information they needed about radiotherapy treatment (young women (35–44) OR_adj,_ = 0.73 [0.58, 0.91]) and Black respondents less likely to receive information about test results in understandable way (OR_adj,_ = 0.56 [0.45, 0.71])) to information about patient’s condition and treatment (young women (16–34) (OR_adj,_ = 0.46 [0.30, 0.70]), Asian (OR_adj,_ = 0.28 [0.21, 0.36]), and less affluent respondents (OR_adj,_ = 0.65 [0.52, 0.81]) more likely to respond they did not receive enough information on that regard).

#### Patients’ distrust of healthcare professionals

There was evidence of distrust of doctors among all young females (particularly aged 16-34 (OR_adj,_ = 0.53 [0.39, 0.72]), Asian (OR = 0.75 [0.60, 0.94]), Black (OR_adj,_ =  0.67 [0.50, 0.91]) and White Other (OR_adj,_ =  0.71 [0.59, 0.87]) respondents. Conversely, females aged 75+ were more likely to respond they had confidence in, and trust in all doctors treating them (OR_adj,_ = 1.25 [1.05, 1.48])). Similarly, findings showed evidence of distrust of nurses. That is, young females (16–34 OR_adj,_ =  0.44 [0.34, 0.57]), Asian (OR_adj,_ =  0.75 [0.62, 0.91]) and Black respondents (OR_adj,_ = 0.68 [0.52, 0.88]) were more likely to report lack of confidence and trust in ward nurses. Conversely, females aged 75+ (OR_adj,_ = 1.29 [1.13.0.57]) and the least affluent females (OR_adj,_ =  1.25 [1.10, 1.42]) more often responded they had more confidence in and trust in all nurses.

#### Lack of tailored services

Findings suggest a lack of tailored and sensitive care, for instance, about privacy (all young females, particularly those aged 16–34 (OR_adj,_ = 0.48 [0.35, 0.65]) less likely to perceive they receive enough privacy to discuss their condition and treatment). Moreover, all young respondents (e.g., 16–34 OR_adj,_ = 0.48 [0.39, 0.60]) and all minority ethnic groups (e.g., White Other OR_adj,_ = 0.61 [0.42, 0.82]), were less likely to feel their views were taken into account when discussion their treatments. Similarly, these groups were more likely to perceive they had not received the best possible care from all the staff (young 16–34 (OR_adj,_ = 0.52 [0.43, 0.64])), Asian (OR_adj,_ = 0.67 [0.58, 0.77]), Black (OR_adj,_ = 0.74 [0.60, 0.90] respondents).

### Candidacy Framework

Through a candidacy lens, we identified individual, interpersonal, and system factors as possible drivers of inequity in breast care experience and outcomes. Figure 3 provides a conceptual framework of how these multidimensional factors operate at each level of the breast cancer system.

We subsequently mapped our findings along the cancer care pathway to depict where these inequities emerged and to locate overlapping themes that exposed respondents to cumulative experiences of inequity (Fig. 4).

## Discussion

In this study, we specifically focused on women with breast cancer to understand their unique experiences along the care pathway and how these related to breast cancer outcomes. To this end, we used a national survey to summarise patterns of inequity in women’s experiences accessing a publicly funded healthcare system in England. We mapped pathways to cancer outcomes and depicted multilevel factors influencing differences in breast cancer care experience.

Firstly, our findings showed marked variation in patient experience across all domains of care. Young women and women of Asian, Black, and Mixed ethnicities descent were less likely to rate their care positively compared to older (65–74 years old) and White British women, respectively. These findings corroborate other evidence suggesting that heterogeneity of experience exists among women with breast cancer [4, 35]. In addition, they reinforce the view that women’s health, particularly cancer in women, requires attention and women should not be homogenised as a single group in research if we are to improve our understanding of their unique needs [5]. Moreover, with the projected global increase in burden from the disease [36], policymakers and providers should be cognisant of, and responsive to the needs of diverse female populations to ensure they provide optimal, equitable, and high-quality cancer care for all women.

Secondly, through a candidacy lens, we identified multiple and cumulative opportunities that influence different experiences of cancer care along the pathway, and we mapped where these differences emerged - from the decision to seek help in the first instance, through to the type of resources (both practical and emotional) that were made available once a diagnosis was received (Fig. 4). Furthermore, we depicted the individual, interpersonal, and system-level factors that may explain inequities in cancer care experience (Fig. 3). Monitoring and evaluating the quality of cancer care through patient experience supports policymakers and providers to identify gaps and areas of improvement and it is a way to make them accountable to the patients they serve [10]. Therefore, our findings provide novel insights about the differences in care experience and help to indicate where healthcare systems should focus their efforts to improve the quality of care for all women.

For instance, at the individual level, we observed prolonged help-seeking intervals after respondents from the younger and minoritised groups (particularly Asian respondents) identified potential symptoms. This finding has important implications as evidence suggests that late presentation results in receiving more advanced stages of diagnosis for both groups [37, 38]. For young women, prolonged help-seeking behaviours may be related to lower risk perception (breast cancer is considered an older women’s disease) and lower breast awareness (lack of understanding of how to recognise symptoms) [39]. Some actions that would improve health-seeking behaviours among young women include increasing symptom awareness and understanding how to breast self-check [37]. For women from minoritised groups it may be related to historical trauma and lack of trust in Western medicine [40], cancer fear and fatalism [41], and perception that cancer is a ‘White women’s disease’ [42]. For these groups, rebuilding trust between communities and the healthcare system is key alongside targeted campaigns to raise awareness of the risk of breast cancer and to improve recognition of symptoms among Black and Asian women [43, 44].

At the interpersonal level, we identified inequities in communication between patients and healthcare professionals, particularly among young, Asian, and Black women. This finding is consistent with previous evidence suggesting that communication influences the way minority ethnic group rate their care [45]. Conversely, effective patient-healthcare professional communication has been associated with better patient outcomes including better adherence, self-management, and quality of care [46]. Strategies to improve communication with minority ethnic groups include providing care with cultural humility, being trustworthy, and increasing shared-decision making [47]. Furthermore, some respondents (particularly younger, Asian, and the most deprived respondents) experienced referral delays from primary to secondary care potentially impacting early diagnosis. This instance could be explained by healthcare professionals’ gatekeeper role and how their judgement may influence patients’ progression through services [48]. Reasons that may influence professionals’ judgement include breast cancer being rare in young women [37], lack of knowledge of how breast cancer symptoms manifest in women from minoritised groups [49], and greater complexity to making referrals in areas of higher poverty [50]. Reducing longer diagnosis intervals and poorer outcomes for women with cancer may be achieved by implementing targeted interventions to improve early diagnosis via primary care referrals [51] and diversifying the medical curriculum to equip professionals with knowledge and confidence to delivery cross-cultural care [44].

At the system level, we identified other inequities that may be explained by operational and logistical challenges embedded in the system. For instance, we identified inequities in receiving a named clinical nurse specialist. When patients received a named clinical nurse specialist, young and minority ethnic groups experienced more barriers to accessing them, and young and minority ethnic groups experienced more communication barriers. There are different explanations for these findings including workforce shortfall [52], complex systems to access healthcare professionals [52], or patients being unaware of entitlement [49]. Since it is widely recognised that clinical nurse specialists play a fundamental role in improving patients’ care and outcomes through the provision of psychological support, treatment management, and care coordination [34, 53, 54], efforts should be made to ensure healthcare systems have adequate capacity and provide equal access to CNSs for all breast cancer patients.

Finally, our findings suggest that breast cancer services are not adequately tailored to the needs of young women and women from minority ethnic groups. This is in line with recent evidence that indicates that health systems do not easily cater to the needs of the diverse populations they serve, and this is apparent for women with cancer [4, 44]. The healthcare system in the UK is based on equity and free at the point of care, therefore all women with breast cancer should receive the same level of care. Despite these premises, these findings indicate that universal services are insufficient to warrant equity of care. Other countries with comparable healthcare systems may experience similar challenges to those identified in this study. Therefore, our findings and recommendations will be valuable for stakeholders beyond the UK.

### Strengths and limitations

Apart from the large national sample size (*n* = 25,408), a key strength of this study is the conceptual framework. Guided by the Candidacy Framework, we informed a deeper and holistic understanding of issues influencing the existing equity gap in accessing and receiving breast cancer care. In addition, we could map pathways to breast cancer outcomes and formulate recommendations to improve the quality of services and experience of breast cancer care. It is worth noting that we focused on the patient journey as self-reported by respondents to a nationwide survey. Therefore, we could not ascertain what factors may have influenced practitioners’ behaviours and decision-making. Neither could we ascertain why respondents may have resisted/declined offers from healthcare professionals or whether other socio-cultural factors may have influenced their decisions and experiences.

The national survey itself has also certain weaknesses that should be considered in interpretations. For instance, minority groups (e.g., minority ethnic groups and sexual minorities) were under-represented in the national datasets [20, 55]. Incidence of breast cancer is lower among minority ethnic groups in the UK compared to White British women and this may explain differences in the sample [56]. Furthermore, we could not ascertain the reasons for non-response and are cognisant that this issue could introduce non-response bias. However, the observed differences are concordant with other studies generally suggesting that minority ethnic groups and minority sexual groups more often report less favourable cancer care experience compared to their White British and heterosexuals, respectively [26, 57]. There was relatively small difference in care experience by socio-economic group across all survey questions. Although, there was not consistency in the direction of this effect, our findings corroborate previous evidence [15]. In addition, it was not possible to assess the effect of disability and comorbidity on care experience among respondents due to the large proportion of missing data. Therefore, the needs of these groups remain unknown. These findings suggest that despite the potential patient care experience surveys have, lack of data completeness translates into systematic missed opportunities to inform quality improvement plans and reduce the equity care gap. Moreover, lack of representation in cancer care experience surveys renders some groups invisible [58] and might perpetuate inequities if services are continuously informed by feedback from the dominant groups in society.

It should also be noted that the proportion of respondents rating their care varied by question and not all questions applied to all participants. This could introduce issues of within-respondent variation in responses across questions. However, previous work found statistically significant average associations between cancer diagnosis and patient experience across all survey questions suggesting that this effect could be expected to be minimal [15]. Furthermore, it is plausible that the differential in length since diagnosis influenced how respondents recalled and rated their experience. However, previous studies identified minimal variation when the analysis was restricted to time since diagnosis [59]. Respondents’ age might have also influenced how they rated their care. For example, older patients (aged ≥75 years) might have rated their care more favourably due to a lack of access to free healthcare by previous generations (known as the ‘gratitude bias’) [16, 60]. While our findings are consistent with previous evidence [15, 61], qualitative work would be necessary to explore the reasons for this intergenerational variation in care experience.

The datasets used in this study did not include individual socioeconomic data and therefore we used an area-level indicator as a proxy measure. We acknowledge that combining individual and area-level measures may pose some challenges to understanding individual-level social risks and caution should be taken when interpreting the results [62]. However, indices of multiple deprivation are validated measures used globally in healthcare research to examine socioeconomic inequities and provide valuable information about communities [63, 64]. For these reasons, the area-level proxy measure was deemed appropriate for this study. In doing so, we observed that our findings are concordant with previous studies that analysed similar datasets and used the same area-level measures [15, 25, 26].

Finally, it is worth mentioning that we used the 2017 and 2018 national datasets for our analysis because the survey was halted during the COVID-19 pandemic and more recent data was not available for analysis at the time the study was undertaken. Evidence suggests the pandemic severely affected cancer services and exacerbated inequities among cancer patients [65–67]. Therefore, it is plausible that inequities in patient experience of breast cancer care reported in this study were greater during the pandemic and continue in the current time. With the growing burden of cancer and the disproportionated impact on minoritised populations [68], our findings illuminate ongoing system challenges. Evidence suggests that a restoration of cancer services to pre-pandemic level is unlikely to ameliorate these challenges unless action is taken, including following a data-driven approach to inform service redesign and prioritisation of services [69]. Therefore, our findings will be relevant to policymakers, providers, and patients beyond the UK, despite the data being collected in 2017 and 2018.

## Conclusion

By focusing on women with breast cancer, our study amplifies previous evidence suggesting that women’s health, particularly cancer in women, requires attention to understand and tailor cancer services to meet their unique needs [4]. Our findings suggest that heterogeneity of experience exists among women with breast cancer and this is partly determined by their differing location in society – for instance, resulting from lived experience at the intersection of gender and age, and gender and ethnicity in relation to structures of power such as the healthcare system [5, 44]. To this end, we reported inequities in patient care experience along the breast cancer pathway in England. We identified that young women and women of Asian, Black, and Mixed ethnicities descent were less likely to rate their care positively across all domains of the continuum of care. It is therefore plausible that similar inequities based on women’s unequal location in society are also present in other countries.

Finally, in this study, we demonstrated how the Candidacy Framework can be used to provide a deeper and holistic understanding of patient cancer experience surveys. In doing so, we reported multilevel factors and overlapping themes that required attention to improve clinical practice and to ensure cancer health systems are responsive to women’s needs. Opportunities for intervening include increasing patients’ agency to seek help early, empowering patients to feel confident to navigate the care pathway, removing barriers, and improving patients’ progression through cancer services. Moreover, to enable high-resolution mapping of inequities in women’s health, governments and organisations should fund a more inclusive and robust data collection systems. Cancer care experience surveys are increasingly used to inform services and make policymakers and providers accountable to the population they serve. Therefore, our approach to assessing these surveys and using findings to inform policy and practice may be valuable to other countries using similar tools.

## Supplementary information

Supplementary Table S1

Supplementary Table S2

Supplementary Table S3

Supplementary Table S4

## Data Availability

The National Cancer Patient Experience Survey (NCPES) data analysed during the current study are available from NHS England, on request through the UK Data Service. The NCPES Survey was conducted by Quality Health on behalf of NHS England.
