# Supplementary material for: Using the Candidacy Framework to understand individual, interpersonal, and system level factors driving inequities in women with breast cancer: a cross-sectional study
Source: BJC Rep. 2024 Oct 23;2:83. doi: 10.1038/s44276-024-00103-4 (PMC11524000; doi:10.1038/s44276-024-00103-4)
Supplement: Supplementary file 1 — Supplementary Table S1 [file 44276_2024_103_MOESM1_ESM.docx]

| **Supplementary Table S1. Questions, items response, and score for all questions** | | | | |
| --- | --- | --- | --- | --- |
| Domain of care | Question number | **Question** | **Number of informative responses** | **N (%) of respondents reporting a positive experience** |
| Seeing your GP | 1 | Saw GP once/twice before being told had to go to hospital | 15,520 | 14,593 (94.0) |
|  | 2 | Patient thought they were seen by a hospital doctor as soon as necessary | 24,951 | 22,380 (89.7) |
|  | 3 | % Saw a GP/other doctor in less than 3 months since patient thought something was wrong | 22,940 | 20,941 (91.3) |
| Diagnostic test | 5 | Staff gave complete explanation of purpose of test(s) | 22,254 | 21,109 (94.9) |
|  | 6 | Patient thought they had their test(s) as soon as necessary | 22,512 | 20,583 (91.4) |
|  | 7 | Given complete explanation of test results in understandable way | 22,506 | 18,603 (82.7) |
| Finding out what was wrong with you | 8 | Patient told they could bring a family member/friend when first told they had cancer | 23,121 | 19,225 (83.1) |
|  | 9 | Patient felt they were told sensitively that they had cancer | 24,961 | 22,139 (88.7) |
|  | 10 | Patient completely understood the explanation of what was wrong | 25,202 | 19,481 (77.3) |
|  | 11 | Patient given written information about the type of cancer they had | 22,636 | 17,399 (76.9) |
| Deciding the best treatment for you | 12 | Patient given a choice of different types of treatment | 23,051 | 19,370 (84.0) |
|  | 13 | Possible side effects explained in an understandable way | 24,762 | 18,535 (74.9) |
|  | 14 | Patient offered practical advice and support in dealing with side effects | 24,648 | 17,036 (69.1) |
|  | 15 | Patient given advice on future side effects | 23,835 | 13,316 (55.9) |
|  | 16 | Patient thinks that their views were taken into account when discussing treatment | 24,848 | 19,532 (78.6) |
| Clinical nurse specialist | 17 | Patient given the name of the CNS in charge of their care | 24,614 | 23,262 (94.5) |
|  | 18 | Patient finds it easy to contact their CNS | 21,042 | 17,939 (85.3) |
|  | 19 | Get understandable answers to important questions all/most of the time (CNS) | 20,447 | 18,023 (88.1) |
| Support for people with cancer | 20 | Hospital staff gave information about support/self-help groups | 22,039 | 19,988 (90.7) |
|  | 21 | Hospital staff gave information about impact of cancer on day activities | 19,087 | 16,405 (85.9) |
|  | 22 | Hospital staff gave information on getting financial help | 13,698 | 8,768 (64.0) |
|  | 23 | Hospital staff told patient they could get free prescriptions | 14,708 | 12,090 (82.2) |
| Operations | 25 | Patient given all information they need about the operation | 19,209 | 18,614 (96.9) |
|  | 26 | Staff explained how operation had gone in understandable way | 19,038 | 14,956 (78.6) |
| Hospital care as an inpatient | 28 | Doctors *and* nurses did not talk in front of patients as if they were not there | 16,933 | 15,138 (89.4) |
|  | 29 | Patient had confidence and trust in all doctors treating them | 16,972 | 14,678 (86.5) |
|  | 30 | Patient's family/someone else close definitely had opportunity to talk to doctor | 13,503 | 10,222 (75.7) |
|  | 31 | Patient had confidence and trust in all ward nurses | 16,915 | 13,109 (77.5) |
|  | 32 | Always / nearly always enough nurses on duty | 16,781 | 11,746 (70.0) |
|  | 33 | Hospital staff asked what name the patient preferred to be called by | 16,595 | 10,537 (63.5) |
|  | 34 | Always given enough privacy when discussing condition/treatment | 16,893 | 14,690 (87.0) |
|  | 35 | Patient was able to discuss worries and fears with staff | 12,688 | 7,039 (55.5) |
|  | 36 | Hospital staff definitely did everything to help control pain | 14,978 | 12,968 (86.6) |
|  | 37 | Always treated with respect and dignity by staff | 16,914 | 15,089 (89.2) |
|  | 38 | Given clear written information about what should / should not do post discharge | 16,087 | 14,740 (91.6) |
|  | 39 | Staff told patient who to contact if worried post discharge | 16,363 | 15,771 (96.4) |
| Hospital care as a day patient/  outpatient | 41 | Day patient/outpatient was able to discuss worries and fears with staff | 20,115 | 14,149 (70.3) |
|  | 42 | Doctor had the right notes and other documentation with them | 23,034 | 22,038 (95.7) |
|  | 44 | Patient given all the information they need about radiotherapy treatment | 13,111 | 11,517 (87.8) |
|  | 45 | Patient given information about whether radiotherapy treatment was working in untestable way | 11,222 | 6,697 (59.7) |
|  | 47 | Patient given all the information they need about chemotherapy treatment | 13,173 | 10,939 (83.0) |
|  | 48 | Patient given information about whether chemotherapy treatment was working in untestable way | 11,727 | 7,364 (62.8) |
| Home care and support | 49 | Family/someone close given all information needed to help care at home | 20,618 | 12,063 (58.5) |
|  | 50 | During cancer treatment, patient received enough care and support from health/social services | 12,710 | 6,831 (53.7) |
|  | 51 | After cancer treatment, patient received enough care and support from health/social services | 7,724 | 3,224 (41.7) |
| Care from your general practice | 52 | GP given enough information about patient's condition and treatment | 20,812 | 19,954 (95.9) |
|  | 53 | GPs and nurses definitely did everything they could to support patient during cancer treatment | 16,738 | 10,013 (59.8) |
| Your overall NHS Care | 54 | Patients received the best possible care from all staff | 24,437 | 14,824 (60.7) |
|  | 55 | Patient was offered a care plan | 18,940 | 7,176 (37.9) |
|  | 56 | Overall, patient thought the administration of their care was good/very good | 25,110 | 22,682 (90.3) |
|  | 57 | Overall, patient felt the wait for attending clinical and appointments for their cancer treatment was about right | 24,953 | 16,774 (67.2) |
|  | 58 | Patient asked if they would like to take part in cancer research | 23,869 | 7,665 (32.1) |
|  | 59 | Overall rating of care | 24,742 | 23,551 (95.2) |
| Signposting (uninformative) questions were excluded from the analysis ( (Q4, Q24, Q27, Q40, Q43, Q46). | | | | |
