# Supplementary material for: Using the Candidacy Framework to understand individual, interpersonal, and system level factors driving inequities in women with breast cancer: a cross-sectional study
Source: BJC Rep. 2024 Oct 23;2:83. doi: 10.1038/s44276-024-00103-4 (PMC11524000; doi:10.1038/s44276-024-00103-4)
Supplement: Supplementary file 2 — Supplementary Table S2 [file 44276_2024_103_MOESM2_ESM.docx]

| **Supplementary Table S2. Summary of sociodemographic characteristics included in the analysis** | | |
| --- | --- | --- |
| **Covariate** | **Definition** | **Categories** |
| Gender | Female hospital record based on sex assigned at birth. | Only patients with a female hospital record were eligible. |
| Age | Age is measured by years from birth.  Age was categorised into six groups in line with other studies and to enable comparison with existing evidence. | [16-34]  [35-44]  [45-54]  [55-64]  [65-74]  [75+] |
| Ethnicity | Self-reported by patients in response to NCPES question 69 – *What is your ethnic group?*, categorised into five broader groups and 18 subgroups (based on the NHS Digital requirements^1^ and informed by the UK Office of National Statistics (ONS) categorisation^2^).  In this present study, the White group was recoded into two groups a) White British (the largest group taken as reference group), and b) Other White (Irish, Gypsy or Irish Traveller, or any other White background). Due to the small sample size, we used Asian (included Indian, Pakistani, Bangladeshi, Chinese, and any other Asian background), Black (included African, Caribbean, and any other Black/Caribbean background), Mixed ethnicity (included White and Black Caribbean, White and Black African, White and Asian, any other mixed/multiple ethnic background) and Other Ethnic groups (included Arab, any other ethnic group) as broader group for analysis. | White British  Other White  Asian  Black  Mixed and other ethnicities. |
| Indices of Multiple Deprivation (IMD) | The IMD is the official composite measure of area-level deprivation in the UK.^3^ The IMD quintiles divide areas into five quintiles on relative disadvantage (1 – the most deprived/the least affluent; 5 – the least deprived/most affluent) and are often used in research. We use this measure because individual socioeconomic data was not included in the 2017/2018 surveys. | 5 (most affluent)  4  3  2  1 (least affluent) |
| Sexual orientation | Self-reported by patients in response to question NCPES question 65 – *Which of the following options best describes how you think of yourself?* Responses were categorised into five groups (heterosexual or straight; gay or lesbian; bisexual; other; prefer not to say).  Due to the small sample size, we recoded sexual orientation into a binary variable a) Heterosexual, and b) Sexual minorities (gay or lesbian, bisexual, other). Non-informative responses (‘prefer not to say’) were excluded from the analysis. | Heterosexuals  Sexual minority group |
