# Supplementary material for: Using the Candidacy Framework to understand individual, interpersonal, and system level factors driving inequities in women with breast cancer: a cross-sectional study
Source: BJC Rep. 2024 Oct 23;2:83. doi: 10.1038/s44276-024-00103-4 (PMC11524000; doi:10.1038/s44276-024-00103-4)
Supplement: Supplementary file 3 — Supplementary Table S3 [file 44276_2024_103_MOESM3_ESM.docx]

| **Table S3. Supplementary Table S3. NCPES survey questions mapped across the stages of the Candidacy Framework** | | | | | | | | | |
| --- | --- | --- | --- | --- | --- | --- | --- | --- | --- |
| **Domain of care** | **Question number** | **Questions** | **Candidacy Framework** | | | | | | |
|  |  |  | **Identification**  **of candidacy** | **Navigation**  **of services** | **Permeability**  **of services** | **Appearance**  **at services** | **Adjudication**  **by HCPs** | **Offers of/**  **resistance to services** | **Operating and local production** |
| **Seeing your GP** | **1** | Saw GP once/twice before being told had to go to hospital | **x** | **x** | **x** | **x** | **x** | **x** | **x** |
|  | **2** | Patient thought they were seen by a hospital doctor as soon as necessary |  |  | **x** |  | **x** |  |  |
|  | **3** | % Saw a GP/other doctor in less than 3 months since patient thought something was wrong | **x** |  |  | **x** |  |  |  |
| **Diagnostic Tests** | **5** | Staff gave complete explanation of purpose of test(s) |  |  | **x** |  | **x** |  |  |
|  | **6** | Patient thought they had their test(s) as soon as necessary |  |  | **x** |  | **x** |  | **x** |
|  | **7** | Given complete explanation of test results in understandable way |  |  | **x** |  | **x** |  |  |
| **Finding what is wrong with you** | **8** | Patient told they could bring a family member/friend when first told they had cancer |  |  | **x** |  |  |  |  |
|  | **9** | Patient felt they were told sensitively that they had cancer |  |  | **x** |  |  |  |  |
|  | **10** | Patient completely understood the explanation of what was wrong |  |  | **x** |  |  |  |  |
|  | **11** | Patient given written information about the type of cancer they had |  |  | **x** |  | **x** |  |  |
| **Deciding the best treatment for you** | **12** | Patient given a choice of different types of treatment |  |  | **x** |  | **x** |  |  |
|  | **13** | Possible side effects explained in an understandable way |  |  | **x** |  |  |  |  |
|  | **14** | Patient offered practical advice and support in dealing with side effects |  |  | **x** |  | **x** | **x** |  |
|  | **15** | Patient given advice on future side effects |  |  | **x** |  | **x** |  |  |
|  | **16** | Patient thinks that their views were taken into account when discussing treatment | **x** |  | **x** |  | **x** |  |  |
| **Clinical nurse specialist** | **17** | Patient given the name of the CNS in charge of their care | **x** | **x** | **x** |  | **x** | **x** | **x** |
|  | **18** | Patient finds it easy to contact their CNS |  | **x** | **x** |  |  |  | **x** |
|  | **19** | Get understandable answers to important questions all/most of the time (CNS) |  |  | **x** |  |  |  |  |
| **Support for people with cancer** | **20** | Hospital staff gave information about support/self-help groups |  |  | **x** |  | **x** | **x** |  |
|  | **21** | Hospital staff gave information about impact of cancer on day activities |  |  | **x** |  | **x** |  |  |
|  | **22** | Hospital staff gave information on getting financial help |  |  | **x** |  | **x** | **x** |  |
|  | **23** | Hospital staff told patient they could get free prescriptions |  |  | **x** |  | **x** | **x** |  |
| **Operations** | **25** | Patient given all information they need about the operation |  |  | **x** |  | **x** |  |  |
|  | **26** | Staff explained how operation had gone in understandable way |  |  | **x** |  |  |  |  |
| **Hospital care as an inpatient** | **28** | Doctors *and* nurses did not talk in front of patients as if they were not there |  |  | **x** |  |  |  |  |
|  | **29** | Patient had confidence and trust in all doctors treating them |  |  | **x** |  |  |  |  |
|  | **30** | Patient's family/someone else close definitely had opportunity to talk to doctor |  |  | **x** |  |  |  |  |
|  | **31** | Patient had confidence and trust in all ward nurses |  |  | **x** |  |  |  |  |
|  | **32** | Always / nearly always enough nurses on duty |  |  |  |  |  |  | **x** |
|  | **33** | Hospital staff asked what name the patient preferred to be called by |  |  | **x** |  |  |  |  |
|  | **34** | Always given enough privacy when discussing condition/treatment |  |  | **x** |  |  |  |  |
|  | **35** | Patient was able to discuss worries and fears with staff |  |  | **x** |  |  |  | **x** |
|  | **36** | Hospital staff definitely did everything to help control pain |  |  | **x** |  | **x** | **x** |  |
|  | **37** | Always treated with respect and dignity by staff |  |  | **x** |  |  |  |  |
|  | **38** | Given clear written information about what should / should not do post discharge | **x** | **x** | **x** |  | **x** | **x** |  |
|  | **39** | Staff told patient who to contact if worried post discharge |  |  | **x** |  | **x** | **x** |  |
| **Hospital care as a day patient/**  **outpatient** | **41** | Day patient/outpatient was able to discuss worries and fears with staff |  |  | **x** |  |  |  | **x** |
|  | **42** | Doctor had the right notes and other documentation with them |  |  | **x** |  |  |  | **x** |
|  | **44** | Patient given all the information they need about radiotherapy treatment | **x** |  | **x** |  | **x** | **x** |  |
|  | **45** | Patient given information about whether radiotherapy treatment was working in untestable way |  |  | **x** |  | **x** | **x** |  |
|  | **47** | Patient given all the information they need about chemotherapy treatment | **x** |  | **x** |  | **x** | **x** |  |
|  | **48** | Patient given information about whether chemotherapy treatment was working in untestable way |  |  | **x** |  | **x** | **x** |  |
| **Home care and support** | **49** | Family/someone close given all information needed to help care at home |  |  | **x** |  | **x** | **x** |  |
|  | **50** | During cancer treatment, patient received enough care and support from health/social services |  |  | **x** |  | **x** | **x** | **x** |
|  | **51** | After cancer treatment, patient received enough care and support from health/social services |  |  | **x** |  | **x** | **x** | **x** |
| **Care from your general practice** | **52** | GP given enough information about patient's condition and treatment |  |  |  |  | **x** |  | **x** |
|  | **53** | GPs and nurses definitely did everything they could to support patient during cancer treatment |  |  | **x** |  | **x** | **x** | **x** |
|  | **54** | Patients received the best possible care from all staff |  |  |  |  | **x** |  | **x** |
|  | **55** | Patient was offered a care plan |  |  | **x** |  | **x** | **x** | **x** |
|  | **56** | Overall, patient thought the administration of their care was good/very good |  |  | **x** |  | **x** | **x** | **x** |
|  | **57** | Overall, patient felt the wait for attending clinical and appointments for their cancer treatment was about right |  |  | **x** | **x** | **x** | **x** | **x** |
|  | **58** | Patient asked if they would like to take part in cancer research |  |  |  |  | **x** | **x** |  |
|  | **59** | Overall rating of care |  |  |  |  |  |  |  |
| Signposting (uninformative) questions were excluded from the analysis (Q4, Q24, Q27, Q40, Q43, Q46). HCPs: Healthcare professionals | | | | | | | | | |
