# Supplementary material for: Using the Candidacy Framework to understand individual, interpersonal, and system level factors driving inequities in women with breast cancer: a cross-sectional study
Source: BJC Rep. 2024 Oct 23;2:83. doi: 10.1038/s44276-024-00103-4 (PMC11524000; doi:10.1038/s44276-024-00103-4)
Supplement: Supplementary file 4 [file 44276_2024_103_MOESM4_ESM.docx]

Supplemental Table S4. Association between respondents socio-demographic characteristics and cancer patient experience (Presenting ORs and 95%CIs).

Q	Items	Age (Reference group: 65-74)					Ethnicity (Reference group: White British)					Socioeconomic position (Reference group: Most affluent (IMD=1)				Sexual orientation (Reference group: Heterosexuals)
		16-34 (OR, 95%CI)	35-44 (OR, 95%CI)	45-54 (OR, 95%CI)	55-64 (OR, 95%CI)	75+ (OR, 95%CI)	White Other (OR, 95%CI)	Mixed (OR, 95%CI)	Asian (OR, 95%CI)	Black (OR, 95%CI)	Other ethnicities (OR, 95%CI)	Least affluent (IMD=1) (OR, 95%CI)	2 (OR, 95%CI)	3 (OR, 95%CI)	4 (OR, 95%CI)	SMG (OR, 95%CI)
1	Saw GP once/twice before being told had to go to hospital	0.74 (0.50,1.09)	1.26 (0.97,1.62)	**1.22 (1.00,1.48)**	1.11 (0.91,1.35)	**1.39 (1.11,1.74)**	0.80 (0.59,1.07)	1.07 (0.53,2.12)	**0.48 (0.36,0.65)**	0.81 (0.51,1.28)	**0.26 (0.15,0.46)**	**0.26 (0.15,0.46)**	0.86 (0.70, 1.07)	1.05 (0.86,1.29)	1.13 (0.92, 1.38)	0.92 (0.54,1.58)
2	Patient thought they were seen by a hospital doctor as soon as necessary	**0.43 (0.36, 0.56)**	**0.44 (0.38,0.51)**	**0.56 (0.50,0.63)**	**0.82 (0.73,0.90)**	**1.30 (1.10,1.53)**	**0.71 (0.60,0.85)**	0.80 (0.54,1.18)	**0.50 (0.41,0.60)**	0.85 (0.63,1.14)	**0.43 (0.29,0.65)**	**1.21 (1.04,1.40)**	1.08 (0.95,1.23)	1.05 (0.93,1.19)	0.99 (0.88,1.11)	0.86 (0.62,1.17)
3	% Saw a GP/other doctor in less than 3 months since patient thought something was wrong	**0.73 (0.52,1.03)**	**0.72 (0.60,0.86)**	**0.82 (0.72,0.94)**	1.05 (0.92,1.20)	**0.68 (0.59,0.78)**	**0.77 (0.62,0.95)**	**0.66 (0.44,0.99)**	**0.68 (0.54,0.86)**	0.74 (0.54,1.01)	0.71 (0.41,1.23)	**0.81 (0.69,0.96)**	0.95 (0.82,1.10)	0.94 (0.82,1.08)	0.99 (0.86,1.13)	0.68 (0.49,0.96)
5	Staff gave complete explanation of purpose of test(s)	**0.62 (0.44,0.88)**	**0.75 (0.61,0.91)**	**0.77 0.66,0.89)**	**1.02** (0.88,1.18)	**0.65 (0.55,0.76)**	**0.79 (0.63,0.99)**	**0.60 (0.39,0.94)**	**0.65 (0.50,0.84)**	**0.69 (0.49,0.97)**	0.91 (0.46,1.76)	**0.82 (0.70,0.98)**	0.95 (0.81,1.16)	0.93 (0.80,1.08)	1.02 (0.88,1.18)	0.76 (0.52,1.11)
6	Patient thought they had their test(s) as soon as necessary	**0.44 (0.32,0.61)**	**0.46 (0.39,0.55)**	**0.55 (0.48,0.62)**	**0.77 (0.67,0.88)**	**1.35 (1.12,1.64)**	**0.77 (0.62,0.94)**	**0.60 (0.40,0.91)**	**0.54 (0.43,0.67)**	1.14 (0.77,1.66)	**0.40 (0.25,0.65)**	1.25 (1.05,1.48)	1.16 (1.00,1.35)	1.07 (0.93,1.23)	1.21 (1.05,1.38)	0.86 (0.60,1.23)
7	Given complete explanation of test results in understandable way	**0.49 (0.38,0.62)**	**0.51 (0.45,0.48)**	**0.67 (0.61,0.74)**	**0.86 (0.78,0.95)**	1.13 (0.99,1.28)	0.89 (0.76,1.10)	0.75 (0.53,1.10)	**0.65 (0.54,0.80)**	**0.56 (0.45,0.70)**	0.80 (0.51,1.30)	0.94 (0.83,1.06)	0.92 (0.83,1.03)	0.92 (0.83,1.02)	0.99 (0.89,1.10)	1.11 (0.82,1.49)
8	Patient told they could bring a family member/friend when first told they had cancer	**0.51 (0.39,0.65)**	**0.56 (0.49,0.65)**	**0.63 (0.57,0.69)**	**0.79 (0.72,0.87)**	**1.21 (1.07,1.38)**	1.08 (0.91,1.30)	**0.68 (0.49,0.95)**	1.05 (0.86,1.28)	0.90 (0.69,1.16)	0.74 (0.49,1.14)	**1.31 (1.15,1.10)**	**1.14 (1.02,1.15)**	1.03 (0.93,1.28)	1.00 (0.90,1.10)	1.14 (0.84,1.56)
9	Patient felt they were told sensitively that they had cancer	**0.53 (0.40,0.69)**	**0.58 (0.50,0.68)**	**0.65 (0.58,0.72)**	**0.71 (0.63,0.79)**	**1.36 (1.16,1.58)**	0.90 (0.75,1.09)	0.46 (0.33,0.64)	0.84 (0.68,1.03)	0.86 (0.65,1.13)	0.58 (0.37,0.90)	**1.27 (1.10,1.47)**	1.12 (0.99,1.26)	**1.15 (1.03,1.29)**	**1.16 (1.04,1.30)**	0.12 (0.61,1.13)
10	Patient completely understood the explanation of what was wrong	**0.42 (0.34,0.52)**	**0.45 (0.40,0.50)**	**0.56 (0.51,0.61)**	**0.74 (0.68,0.80)**	**1.41 (1.26,1.57)**	**1.41 (1.21,1.65)**	0.75 (0.56,1.00)	0.90 (0.77,1.06)	**0.70 (0.57,0.86)**	1.04 (0.70,1.54)	0.95 (0.86,1.11)	0.94 (0.85,1.04)	0.95 (0.87,1.03)	1.02 (0.93,1.06)	0.88 (0.69,1.12)
11	Patient given written information about the type of cancer they had	**0.66 (0.52,0.83)**	**0.68 (0.60,0.77)**	**0.82 (0.75,0.90)**	0.93 (0.85,1.01)	**0.78 (0.70,0.86)**	0.86 (0.74,1.00)	0.77 (0.56,1.05)	**0.57 (0.49,0.66)**	**0.69 (0.55,0.86)**	**0.66 (0.44,0.96)**	0.97 (0.87,1.09)	1.04 (0.94,1.15)	1.00 (0.91,1.10)	0.97 (0.89,1.06)	0.92 (0.71,1.20)
12	Patient given a choice of different types of treatment	**0.45 (0.35,0.58)**	**0.50 (0.43,0.57)**	**0.67 (0.60,0.74)**	**0.83 (0.76,0.92)**	1.11 (0.98,1.27)	1.04 (0.88,1.04)	0.73 (0.52,0.92)	0.83 (0.69,1.00)	0.73 (0.57,1.02)	0.68 (0.45,1.24)	**1.29 (1.14,1.47)**	**1.27 (1.14,1.43)**	1.08 (0.98,1.20)	1.05 (0.95,1.16)	0.95 (1.28,1.28)
13	Possible side effects explained in an understandable way	**1.45 (1.13,1.88)**	1.13 (0.99,1.28)	1.01 (0.93,1.10)	1.00 (0.93,1.08)	1.01 (0.92,1.11)	0.99 (0.85,1.38)	**0.71 (0.53,0.85)**	**0.82 (0.70,0.96)**	**0.69 (0.56,0.96)**	0.92 (0.62,1.14)	**1.13 (1.02,1.25)**	**1.10 (1.01,1.21)**	1.00 (0.92,1.08)	1.02 (0.94,1.11)	0.93 (0.73,1.19)
14	Patient offered practical advice and support in dealing with side effects	**0.80 (0.64,0.99)**	**0.72 (0.64,0.81)**	**0.74 (0.68,0.79)**	**0.89 (0.82,0.96)**	0.97 (0.89,1.07)	0.97 (0.85,1.11)	0.78 (0.59,1.03)	**0.86 (0.74,0.99)**	**0.68 (0.56,0.83)**	0.95 (0.65,1.37)	**1.18 (1.07,1.31)**	**1.12 (1.03,1.23)**	1.03 (0.95,1.11)	1.04 (0.96,1.13)	0.89 (0.71,1.12)
15	Patient given advice on future side effects	1.00 (0.81,1.23)	**0.72 (0.64,0.80)**	**0.74 (0.69,0.79)**	**0.84 (0.78,0.90)**	1.00 (0.91,1.09)	**1.14 (1.00,2.19)**	0.96 (0.73,0.95)	1.11 (0.96,1.29)	**0.78 (0.64,1.26)**	**1.51 (1.04,1.30)**	**1.38 (1.26,1.51)**	**1.19 (1.10,1.30)**	1.02 (0.95,1.10)	**1.09 (1.01,1.17)**	0.95 (0.76,1.91)
16	Patient thinks that their views were taken into account when discussing treatment	**0.48 (0.39,1.20)**	**0.54 (0.48,0.84)**	**0.67 (0.62,0.73)**	**0.77 (0.71,0.62)**	1.08 (0.97,0.60)	**0.61 (0.42,0.88)**	**0.65 (0.53,0.81)**	**0.74 (0.63,0.86)**	**0.73 (0.54,0.98)**	**0.83 (0.72,0.96)**	1.10 (0.98,1.04)	0.99 (0.89,1.07)	0.98 (0.89,1.09)	0.95 (0.87,1.23)	0.80 (0.63,1.03)
17	Patient given the name of the CNS in charge of their care	0.89 (0.56,0.93)	0.84 (0.66,1.34)	0.88 (0.75,1.03)	0.97 (0.84,1.06)	**0.78 (0.65,1.40)**	1.00 (0.75,7.53)	1.08 (0.58,1.56)	1.05 (0.76,1.45)	1.01 (0.65,1.99)	2.37 (0.74,1.32)	**1.32 (1.07,1.63)**	1.05 (0.88,1.24)	**1.26 (1.07,1.47)**	**1.24 (1.06,1.45)**	0.67 (0.44,1.02)
18	Patient finds it easy to contact their CNS	**0.66 (0.50,0.88)**	**0.68 (0.58,0.79)**	**0.77 (0.68,0.85)**	**0.83 (0.75,0.92)**	1.15 (1.00,1.32)	**0.73 (0.62,0.87)**	0.74 (0.51,1.06)	**0.68 (0.56,0.82)**	**0.75 (0.57,0.97)**	0.76 (0.48,1.22)	1.07 (0.93,1.24)	0.99 (0.87,1.24)	0.92 (0.82,1.03)	1.03 (0.92,1.15)	0.85 (0.63,1.16)
19	Get understandable answers to important questions all/most of the time (CNS)	**0.67 (0.50,0.91)**	**0.67 (0.57,0.79)**	**0.79 (0.70,0.89)**	**0.81 (0.72,0.91)**	1.02 (0.87,1.18)	0.86 (0.71,1.05)	**0.54 (0.38,0.77)**	**0.64 (0.52,0.78)**	**0.51 (0.39,0.67)**	**0.52 (0.33,0.83)**	0.93 (0.80,1.09)	0.93 (0.81,1.06)	0.95 (0.83,1.08)	1.00 (0.88,1.13)	0.84 (0.60,1.16)
20	Hospital staff gave information about support/self-help groups	**0.50 (0.37,0.68)**	0.71 (0.59,1.12)	**0.79 (0.69,0.89)**	0.99 (0.87,0.85)	**0.82 (0.70,0.67)**	0.94 (0.75,1.98)	0.79 (0.52,1.52)	**0.73 (0.58,0.91)**	1.08 (0.76,1.21)	1.05 (0.56,1.17)	**0.83 (0.70,0.98)**	0.88 (0.76,1.02)	0.91 (0.79,1.04)	0.88 (0.77,1.01)	0.88 (0.62,1.26)
21	Hospital staff gave information about impact of cancer on day activities	0.87 (0.63,1.18)	**0.72 (0.65,0.82)**	**0.73 (0.65,0.82)**	**0.73 (0.65,0.85)**	0.84 (0.71,1.18)	1.06 (0.86,1.99)	0.74 (0.51,1.01)	0.94 (0.76,1.17)	**0.77 (0.59,1.07)**	1.13 (0.64,1.30)	0.99 (0.86,1.15)	1.03 (0.91,1.18)	0.96 (0.85,1.09)	0.98 (0.87,1.10)	0.81 (0.59,1.10)
22	Hospital staff gave information on getting financial help	1.14 (0.90,1.46)	1.02 (0.89,1.17)	1.05 (0.95,1.17)	1.08 (0.98,1.19)	**0.72 (0.62,0.82)**	0.89 (0.74,1.05)	0.76 (0.55,1.06)	0.90 (0.75,1.07)	**1.26 (1.00,1.06)**	0.88 (0.58,1.33)	0.98 (0.87,1.11)	1.02 (0.91,1.15)	0.97 (0.87,1.09)	0.90 (0.81,1.00)	1.09 (0.83, 1.44)
23	Hospital staff told patient they could get free prescriptions	**0.59 (0.43,0.80)**	**0.46 (0.38,0.55)**	**0.46 (0.39,0.53)**	**0.46 (0.40,0.56)**	1.15 (0.87,1.52)	0.91 (0.76,1.43)	0.74 (0.52,1.36)	0.99 (0.81,1.22)	1.02 (0.77,1.07)	0.89 (0.55,1.10)	1.08 (0.92,1.27)	0.93 (0.81,1.06)	0.94 (0.82,1.07)	**0.86 (0.76,0.98)**	1.00 (0.72,1.40)
25	Patient given all information they need about the operation	1.04 (0.60,1.82)	0.83 (0.63,1.08)	0.87 (0.72,1.05)	0.89 (0.74,1.07)	0.99 (0.78,1.24)	0.75 (0.56,2.64)	0.73 (0.39,2.07)	0.86 (0.60,1.23)	1.18 (0.68,1.37)	1.01 (0.39,1.02)	1.05 (0.83,1.33)	0.97 (0.79,1.19)	1.08 (0.89,1.32)	1.04 (0.86,1.26)	0.99 (0.56,1.75)
26	Staff explained how operation had gone in understandable way	**0.74 (0.57,0.97)**	**0.72 (0.62,0.82)**	**0.74 (0.67,0.81)**	**0.80 (0.73,0.88)**	1.06 (0.94,1.20)	0.97 (0.81,1.15)	1.00 (0.70,1.44)	**0.71 (0.59,0.85)**	0.87 (0.68,1.13)	1.46 (0.84,2.51)	1.05 (0.93,1.19)	1.06 (0.95,1.18)	0.99 (0.90,1.10)	1.08 (0.98,1.19)	1.14 (0.84,1.54)
28	Doctors *and* nurses did not talk in front of patients as if they were not there	**0.53 (0.38,0.72)**	**0.67 (0.56,0.81)**	**0.73 (0.63,0.84)**	**0.83 (0.72,0.96)**	0.87 (0.73,1.03)	**0.55 (0.45,0.68)**	**0.45 (0.30,0.68)**	**0.44 (0.35,0.54)**	0.79 (0.57,1.10)	**0.45 (0.27,0.75)**	**0.61 (0.52,0.72)**	**0.75 (0.64,0.88)**	0.94 (0.81,1.10)	0.98 (0.84,1.14)	**0.60 (0.43,0.83)**
29	Patient had confidence and trust in all doctors treating them	**0.53 (0.39,0.72)**	**0.54 (0.45,0.64)**	**0.65 (0.58,0.74)**	**0.68 (0.60,0.76)**	**1.25 (1.05,1.48)**	**0.71 (0.59,0.87)**	0.87 (0.56,1.36)	**0.75 (0.60,0.94)**	**0.67 (0.50,0.91)**	1.09 (0.59,2.03)	1.01 (0.86,1.19)	0.94 (0.81,1.08)	0.96 (0.84,1.09)	0.91 (0.80,1.03)	0.76 (0.54,1.06)
30	Patient's family/someone else close definitely had opportunity to talk to doctor	**0.46 (0.35,0.60)**	**0.48 (0.41,0.57)**	**0.58 (0.52,0.65)**	**0.72 (0.65,0.81)**	**1.25 (1.08,1.45)**	**0.83 (0.68,0.99)**	**0.63 (0.43,0.93)**	0.86 (0.70,1.05)	1.25 (0.91,1.71)	**1.97 (1.05,3.70)**	**1.17 (1.02,1.35)**	1.10 (0.97,1.25)	0.99 (0.88,1.11)	1.03 (0.92,1.56)	1.02 (0.73,1.42)
31	Patient had confidence and trust in all ward nurses	**0.44 (0.34,0.57)**	**0.58 (0.50,0.67)**	**0.68 (0.61,0.75)**	**0.81 (0.73,0.67)**	**1.29 (1.13,0.57)**	0.96 (0.80,1.15)	0.93 (0.64,1.35)	**0.75 (0.62,0.91)**	**0.68 (0.52,0.88)**	1.28 (0.76,2.18)	**1.25 (1.09,1.43)**	1.08 (0.96,1.21)	1.05 (0.94,1.16)	0.99 (0.88,1.09)	1.04 (0.76,1.41)
32	Always / nearly always enough nurses on duty	**0.39 (0.31,0.50)**	**0.44 (0.39,0.51)**	**0.57 (0.52,0.63)**	**0.75 (0.68,0.82)**	**1.19 (1.05,1.35)**	**1.25 (1.05,1.49)**	1.10 (0.77,1.58)	**0.78 (0.65,0.94)**	1.13 (0.87,1.47)	1.24 (0.77,1.99)	1.00 (0.89,1.13)	1.00 (0.90,1.12)	0.95 (0.86,1.05)	**0.91 (0.82,0.99)**	0.91 (0.69,1.20)
33	Hospital staff asked what name the patient preferred to be called by	**0.48 (0.38,0.61)**	**0.53 (0.46,0.60)**	**0.59 (0.54,0.64)**	**0.74 (0.68,0.81)**	**1.28 (1.14,1.44)**	1.10 (0.93,1.29)	0.80 (0.57,1.12)	**0.78 (0.65,0.93)**	0.80 (0.63,1.03)	0.67 (0.43,1.05)	1.30 (1.16,1.46)	**1.16 (1.04,1.28)**	1.09 (0.99,1.20)	1.02 (0.93,1.11)	0.90 (0.69,1.18)
34	Always given enough privacy when discussing condition/treatment	**0.48 (0.35,0.65)**	**0.52 (0.43,0.62)**	**0.56 (0.50,0.64)**	**0.64 (0.56,0.72)**	**1.39 (1.15,1.68)**	1.25 (0.99,1.58)	0.67 (0.44,1.00)	0.97 (0.76,1.24)	0.90 (0.65,1.24)	0.98 (0.55,1.76)	1.11 (0.94,1.31)	1.09 (0.94,1.25)	1.10 (0.96,1.25)	1.03 (0.91,1.18)	**0.70 (0.50,0.97)**
35	Patient was able to discuss worries and fears with staff	**0.56 (0.43,0.72)**	**0.59 (0.51,68)**	**0.69 (0.63,0.77)**	**0.83 (0.75,0.92)**	0.94 (0.83,1.07)	0.98 (0.82,1.16)	0.97 (0.67,1.41)	0.88 (0.73,1.06)	0.99 (0.76,1.30)	1.60 (0.99,2.59)	**1.25 (1.10,1.42)**	**1.17 (1.05,1.31)**	1.08 (0.97,1.20)	1.05 (0.95,1.16)	1.15 (0.86,1.55)
36	Hospital staff definitely did everything to help control pain	**0.36 (0.27,0.47)**	**0.48 (0.40,0.57)**	**0.61 (0.53,0.69)**	**0.81 (0.70,0.93)**	**1.20 (0.99,1.46)**	0.91 (0.73,1.14)	0.91 (0.57,1.46)	**0.54 (0.44,0.67)**	**0.76 (0.55,1.05)**	0.91 (0.50,1.67)	1.05 (0.89,1.25)	0.99 (0.85,1.15)	0.98 (0.85,1.13)	0.93 (0.81,1.06)	1.14 (0.77,1.67)
37	Always treated with respect and dignity by staff	**0.42 (0.31,0.57)**	**0.51 (0.42,0.61)**	**0.58 (0.50,0.66)**	**0.75 (0.65,0.87)**	**1.37 (1.12,1.67)**	1.29 (1.00,1.68)	0.85 (0.53,1.35)	0.79 (0.62,1.01)	0.83 (0.59,1.16)	3.09 (1.12,8.49)	1.10 (0.92,1.31)	0.96 (0.83,1.12)	1.07 (0.93,1.25)	0.96 (0.83,1.10)	0.90 (0.61,1.32)
38	Given clear written information about what should / should not do post discharge	**0.51 (0.36,0.73)**	**0.69 (0.55,0.86)**	**0.77 (0.66,0.90)**	0.95 (0.81,1.12)	**0.80 (0.66,0.97)**	0.82 (0.63,1.05)	0.79 (0.46,1.34)	1.05 (0.76,1.44)	1.24 (0.78,1.96)	2.98 (0.93,9.50)	1.10 (0.90,1.34)	1.13 (0.94,1.35)	1.00 (0.85,1.18)	0.96 (0.82,1.13)	0.71 (0.47,1.07)
39	Staff told patient who to contact if worried post discharge	**0.62 (0.39,0.98)**	**0.72 (0.55,0.94)**	0.90 (0.73,1.10)	0.98 (0.80,1.19)	0.85 (0.68,1.08)	1.02 (0.72,1.44)	0.79 (0.40,1.55)	0.81 (0.57,1.16)	1.13 (0.65,1.99)	1.11 (0.41,2.96)	0.90 (0.70,1.15)	0.88 (0.70,1.10)	0.98 (0.79,1.21)	0.88 (0.72,1.08)	**0.59 (0.37,0.95)**
41	Day patient/outpatient was able to discuss worries and fears with staff	**0.76 (0.59,0.96)**	**0.63 (0.56,0.71)**	**0.78 (0.71,0.85)**	**0.86 (0.79,0.93)**	1.01 (0.91,1.12)	**0.82 (0.71,0.95)**	0.75 (0.55,1.01)	**0.71 (0.61,0.84)**	0.83 (0.66,1.05)	0.90 (0.60,1.35)	**1.25 (1.12,1.39)**	1.06 (0.97,1.17)	1.04 (0.95,1.14)	1.08 (0.99,1.18)	1.09 (0.84,1.41)
42	Doctor had the right notes and other documentation with them	0.66 (0.44,1.00)	**0.65 (0.52,0.81)**	**0.81 (0.69,0.96)**	0.87 (0.75,1.02)	1.04 (0.85,1.28)	0.90 (0.69,1.18)	0.61 (0.37,1.00)	1.02 (0.74,1.42)	1.00 (0.64,1.57)	1.36 (0.55,3.36)	1.19 (0.97,1.47)	1.11 (0.93,1.33)	1.04 (0.88,1.22)	1.04 (0.88,1.21)	0.78 (0.51,1.20)
44	Patient given all the information they need about radiotherapy treatment	0.87 (0.55,1.37)	**0.73 (0.58,0.91)**	**0.73 (0.63,0.85)**	0.87 (0.75,1.01)	0.84 (0.70,1.01)	**1.38 (1.03,1.84)**	0.67 (0.41,1.10)	1.04 (0.77,1.39)	0.81 (0.56,1.18)	1.58 (0.67,3.66)	1.12 (0.92,1.36)	1.02 (0.86,1.21)	0.93 (0.79,1.08)	0.87 (0.74,1.00)	1.03 (0.66,1.60)
45	Patient given information about whether radiotherapy treatment was working in understandable way	0.73 (0.53,1.01)	**0.76 (0.64,0.89)**	**0.82 (0.73,0.91)**	0.91 (0.82,1.00)	0.95 (0.83,1.08)	**1.65 (1.35,2.00)**	1.01 (0.67,1.51)	**1.42 (1.15,1.76)**	0.83 (0.62,1.10)	**1.83 (1.06,3.12)**	**1.24 (1.09,1.42)**	1.12 (0.99,1.27)	0.95 (0.85,1.07)	0.98 (0.88,1.10)	0.92 (0.68,1.26)
47	Patient given all the information they need about chemotherapy treatment	1.13 (0.81,1.57)	0.86 (0.73,1.06)	0.96 (0.84,1.08)	1.04 (0.91,1.18)	0.86 (0.71,1.03)	1.28 (1.01,1.64)	0.81 (0.52,1.26)	1.00 (0.78,1.28)	0.89 (0.66,1.22)	1.20 (0.65,2.23)	**1.28 (1.08,1.51)**	**1.17 (1.02,1.36)**	1.08 (0.95,1.23)	1.10 (0.97,1.25)	0.77 (0.54,1.09)
48	Patient given information about whether chemotherapy treatment was working in untestable way	0.91 (0.71,1.17)	0.91 (0.79,1.05)	1.02 (0.92,1.13)	1.01 (0.91,1.12)	1.13 (0.97,1.33)	1.29 (1.07,1.56)	0.74 (0.52,1.07)	1.19 (0.97,1.47)	0.90 (0.70,1.15)	1.18 (0.73,1.91)	**1.06 (0.95,1.18)**	1.12 (1.00,1.25)	1.15 (1.02,1.30)	1.23 (1.07,1.40)	1.06 (0.78,1.45)
49	Family/someone close given all information needed to help care at home	**0.66 (0.53,0.81)**	**0.69 (0.61,0.77)**	**0.79 (0.73,0.85)**	**0.84 (0.78,0.91)**	1.08 (0.98,1.19)	1.08 (0.94,1.25)	0.85 (0.63,1.13)	1.16 (0.99,1.35)	0.82 (0.66,1.02)	1.01 (0.69,1.46)	**1.22 (1.10,1.35)**	1.02 (0.94,1.11)	1.01 (0.93,1.10)	1.02 (0.94,1.10)	1.04 (0.82,1.32)
50	During cancer treatment, patient received enough care and support from health/social services	**0.70 (0.54,0.90)**	**0.80 (0.69,0.92)**	**0.89 (0.80,0.98)**	0.91 (0.82,1.00)	0.99 (0.88,1.12)	1.01 (0.85,1.19)	0.70 (0.49,0.99)	0.86 (0.72,1.02)	**0.73 (0.56,0.95)**	0.75 (0.48,1.15)	**0.85 (0.75,0.96)**	**0.83 (0.74,0.93)**	**0.83 (0.75,0.93)**	**0.79 (0.71,0.87)**	0.94 (0.70,1.26)
51	After cancer treatment, patient received enough care and support from health/social services	0.76 (0.55,1.05)	0.88 (0.73,1.05)	**0.84 (0.74,0.96)**	0.95 (0.84,1.09)	1.15 (0.98,1.35)	1.06 (0.86,1.30)	0.86 (0.55,1.32)	0.94 (0.76,1.16)	0.79 (0.57,1.08)	0.85 (0.51,1.42)	0.92 (0.78,1.08)	0.88 (0.76,1.02)	**0.86 (0.75,0.99)**	**0.80 (0.70,0.92)**	0.85 (0.60,1.21)
52	GP given enough information about patient's condition and treatment	**0.46 (0.30.0.70)**	**0.56 (0.43,0.73)**	**0.69 (0.56,0.84)**	**0.81 (0.67,0.99)**	1.20 (0.91,1.58)	**0.42 (0.32,0.54)**	**0.48 (0.28,0.82)**	**0.28 (0.21,0.36)**	0.85 (0.53,1.36)	**0.39 (0.21,0.75)**	**0.73 (0.57,0.94)**	**0.65 (0.52,0.81)**	0.81 (0.65,1.01)	0.94 (0.76,1.17)	0.79 (0.50,1.26)
53	GPs and nurses definitely did everything they could to support patient during cancer treatment	0.80 (0.64,1.01)	0.97 (0.86,1.10)	1.04 (0.95,1.13)	0.99 (0.91,1.08)	1.03 (0.93,1.15)	0.91 (0.78,1.05)	0.95 (0.69,1.30)	**0.82 (0.69,0.96)**	**0.72 (0.58,0.90)**	0.83 (0.56,1.25)	1.00 (0.90,1.12)	0.98 (0.89,1.08)	0.99 (0.90,1.09)	0.98 (0.90,1.07)	1.25 (0.98,1.62)
54	Patients received the best possible care from all staff	**0.52 (0.43,0.64)**	**0.58 (0.52,35)**	**0.71 (0.66,0.76)**	**0.78 (0.73,0.84)**	**1.25 (1.14,1.37)**	**0.79 (0.70,0.90)**	**0.75 (0.57,0.99)**	**0.67 (0.58,0.77)**	**0.74 (0.60,0.90)**	0.75 (0.53,1.07)	**1.19 (1.09,1.31)**	**1.09 (1.00,1.18)**	1.08 (1.00,1.17)	0.99 (0.92,1.06)	0.93 (0.74,1.16)
55	Patient was offered a care plan	**0.63 (0.48,0.81)**	**0.63 (0.55,0.72)**	**0.74 (0.68,0.81)**	**0.83 (0.76,0.89)**	**1.13 (1.03,1.25)**	**1.45 (1.25,1.68)**	0.93 (0.67,1.29)	**1.69 (1.42,2.01)**	**1.32 (1.05,1.66)**	**1.56 (1.02,2.39)**	**1.55 (1.39,1.72)**	**1.29 (1.17,1.41)**	**1.11 (1.02,1.26)**	1.08 (0.99,1.17)	1.27 (0.99,1.63)
56	Overall, patient thought the administration of their care was good/very good	**0.65 (0.47,0.89)**	**0.67 (0.56,0.80)**	**0.72 (0.64,0.82)**	**0.78 (0.70,0.88)**	0.99 (0.86,1.15)	0.98 (0.8,1.21)	0.86 (0.57,1.31)	1.09 (0.85,1.38)	**1.52 (1.04,2.22)**	1.31 (0.70,2.45)	**1.40 (1.19,1.64)**	**1.20 (1.05,1.37)**	1.12 (0.99,1.26)	1.04 (0.93,1.17)	0.90 (0.64,1.27)
57	Overall, patient felt the wait for attending clinical and appointments for their cancer treatment was about right	**0.61 (0.50,0.75)**	**0.63 (0.56,0.70)**	**0.78 (0.72,0.84)**	**0.92 (0.85,0.99)**	**1.24 (1.12, 1.36)**	**0.87 (0.76,0.99)**	**0.74 (0.56,0.98)**	**0.73 (0.63,0.85)**	**0.69 (0.56,0.84)**	0.87 (0.60,1.25)	**1.32 (1.20,1.46)**	**1.10 (1.01,1.20)**	1.03 (0.95,1.11)	**1.11 (1.03,1.20)**	0.96 (0.76,1.20)
58	Patient asked if they would like to take part in cancer research	**1.90 (1.53,2.37)**	**1.72 (1.53,1.93)**	**1.36 (1.26,1.47)**	**1.17 (1.08,1.26)**	**0.81 (0.73,0.89)**	**1.59 (1.39,1.83)**	**1.44 (1.08,1.92)**	**1.20 (1.02,1.41)**	**1.85 (1.50,2.28)**	1.44 (0.97,2.13)	1.02 (0.92,1.11)	1.08 (0.98,1.14)	1.04 (0.96,1.18)	1.02 (0.94,1.23)	1.12 (0.88,1.43)
59	Overall rating of care	**0.56 (0.37,0.86)**	**0.55 (0.44,0.69)**	**0.58 (0.49,0.69)**	**0.75 (0.63,0.89)**	0.88 (0.71,1.09)	**0.63 (0.49,0.81)**	0.80 (0.46,1.40)	**0.52 (0.4,0.67)**	**0.67 (0.46,0.97)**	0.60 (0.32,1.13)	0.83 (0.67,1.03)	0.88 (0.72,1.06)	0.87 (0.72,1.04)	0.91 (0.76,1.08)	0.99 (0.62,1.57)

Data represents Odds Ratio (OR) adjusted for sociodemographic factors and hospital attended for treatment. OR = Odds Ratio. OR >1 indicates higher odds to rate care experience positively compared to the reference group; OR <1 indicates lower odds to rate care experience positively compared to the reference group. CI: Confidence Interval. Bold data represents p-value <0.005. Signposting (uninformative) questions were excluded from the analysis (Q4, Q24, Q27, Q40, Q43, Q46). IMD: Indices of Multiple Deprivation (official composite measure of area-level deprivation in England). SMG: Sexual minority groups Include gay or lesbian, bisexual, and other.
